# Development and Validation of the Chinese Family Resilience Scale in Families in Hong Kong

**DOI:** 10.3390/ijerph20031929

**Published:** 2023-01-20

**Authors:** Janet T. Y. Leung, Daniel T. L. Shek, Chak-Man Tang

**Affiliations:** Department of Applied Social Sciences, The Hong Kong Polytechnic University, Hung Hom, Hong Kong, China

**Keywords:** family resilience, validation, measurement invariance, confirmatory factor analysis, Chinese

## Abstract

Family resilience is an important protective factor mitigating the negative impact of adversities on individuals and families. As there are very few validated measures of family resilience in the Chinese context, we have developed the Chinese Family Resilience Scale (C-FRS) based on the family resilience framework of Froma Walsh. In this paper, we outline the development and the validation of the C-FRS with reference to the socio-cultural context of Hong Kong. Based on content validation involving family experts, the initial 51 items were assessed in terms of their relevance, clarity, and representativeness. The results showed that these items possessed adequate content validity. In order to validate the 51-item measure, we examined its convergent validity, its factorial validity, and its internal consistency based on the responses of 1020 Chinese families (*N* = 2858 participants). After removing the items with unsatisfactory psychometric properties, we retained 35 items in the final scale. The results showed that the C-FRS scores were significantly related to family functioning, thus providing support for its convergent validity. This study also provided support for the conceptual model of Walsh (i.e., three high-order domains involving nine primary indicators). Most importantly, the measurement invariance tests showed that the dimensions of the C-FRS were invariant among fathers, mothers, and adolescent children. As the findings support the reliability and the validity of the 35-item C-FRS, we suggest that this measure can be objectively used to assess family resilience in Chinese communities.

## 1. Introduction

External threats such as economic recession constitute adversities for individuals and families [1,2]. Particularly, the COVID-19 pandemic has brought physical and psychological hazards to individuals and families [3], such as family dysfunctions and disorganization [4,5]. However, some families can overcome the disruptions that are created by adversities, and they also become stronger and more resilient [6,7]. Hence, family scholars have paid special attention to the family processes that protect families from dysfunctions and disorganization under adversity [8,9]. For example, in the family adjustment and adaptation response (FAAR) model, which was proposed by Patterson [10], four family processes (family meaning, cohesion, flexibility, and communication) can help families to cope with family stress and challenges. McCubbin et al. [11] also proposed the resiliency model of family adjustment and adaptation to describe the processes that are involved in family problem solving in response to stresses that are faced by the family. In the family resilience framework, which was proposed by Froma Walsh [9], family resilience includes nine qualitatively distinctive domains subsuming under three broader dimensions. These include family beliefs system (making positive meaning about adversity, a positive outlook, and transcendence and spirituality), organizational patterns (flexibility, connectedness, and social and economic resources), and communication processes (clear information about adversity, open emotional expression, and collaborative problem solving). The framework [9] and the related assessment tool have been widely adopted in family resilience research in the West [12,13].

### 1.1. Research Gaps

Despite the crucial role that family resilience plays in positive psychology and family wellbeing [14], research on family resilience is still in its infancy [15]. One of the hurdles is the severe lack of validated measures that can adequately assess family resilience within a culture. Based on Walsh’s conceptualization of family resilience [14], Rocchi et al. [12] developed and validated the 26-item Italian Walsh family resilience questionnaire in a clinical sample and their relatives. However, only three domains (i.e., shared beliefs and support, family organization and interaction, and the utilization of social resources) were supported by confirmatory factor analysis (CFA). Sixbey [13] developed the 54-item family resilience assessment scale (FRAS), which has been widely adopted and translated into different versions [16,17,18,19,20]. However, only six dimensions (i.e., family communication and problem solving, social and economic resources, a positive outlook, family connectedness, family spirituality, and making a meaning of adversity) were extracted by exploratory factor analysis (EFA) in a sample of 418 respondents in the US. In Chinese societies, the factor structure of the translated Chinese versions of the FRAS is unclear. For instance, in a sample of 502 Chinese primary caregivers of children with developmental delay in Taiwan, Chiu et al. [17] confirmed a six-factor structure that resembled the extracted factor structure in Sixbey’s [13] study. However, Li et al. [20] identified a three-factor structure (i.e., family communication and problem solving, the utilization of social resources, and the maintenance of a positive outlook) using CFA in a sample of 991 university students in China. Besides, in Li et al.’s [20] study, only 32 items out of the 54 items were retained in the measurement, with 22 of the items dropped because of low factor loadings (<0.40) or poor representations of the subscales, with three of the subscales dropped eventually. In a sample of 323 Chinese family caregivers in Hong Kong, Chu et al. [18] identified a five-factor structure (i.e., the ability to make a meaning of adversity, family communication and problem solving, family spirituality, maintaining a positive outlook, and utilizing social and economic resources), with 12 items dropped from the analysis. The inconsistency of the findings on the factor structure across the studies may be due to the employment of different scales, different informants, and/or the utilization of translated measures.

From a cross-cultural perspective, there are challenges regarding the direct borrowing of family measures that are developed in Western societies [21] because there are different family beliefs and processes in families in different cultures [22]. While the Western conceptualization of family processes focuses mainly on individualism and independence, the Chinese view emphasizes the importance of collectivism and interdependence [23].

Another problem in the existing studies is that the sample size is generally small, which suggests that the findings that are based on factor analyses may not be stable. In Chew and Haase’s [16] study of a sample of young people with epilepsy in Singapore, the sample size was 152. In Chu et al.’s [18] study, EFA and CFA were analyzed in two samples of 150 and 173 caregivers, respectively. Leone, Dorstyn, and Ward’s [19] study was based on a sample of 155 caregivers who had a child suffering from neurodevelopmental disorders. Moreover, most of the studies employed caregivers of family members with difficulties (e.g., [17,18,19]), while studies using community samples are few. Obviously, while it is important to examine the psychometric properties of family resilience measures in clinical samples, validation research that is based on community samples is equally as important [24]. The small sample size also suggests that the basic assumptions of factor analysis are not adequately met.

Besides, another weakness of the existing studies is that researchers commonly use a single source of informants, which is mostly the caregivers [12,17,18]. However, based on family systems theories [25,26], fathers, mothers, and children are important members of the family, and they are interdependent. Hence, studies using multiple informants can give a comprehensive picture of the family processes and dynamics within a family.

Finally, most of the existing studies were conducted in a non-pandemic context. As COVID-19 is stressful for families [27,28], there is a need to understand family resilience under COVID-19.

### 1.2. The Current Study

In response to the above-mentioned research gaps, this current study has attempted to develop and validate a family resilience measure of Chinese families under pandemic conditions based on the family resilience framework of Walsh [9]. There are two phases of this study. The first phase involved the development of the family resilience scale for Chinese families. After developing the initial item pool, we conducted content validation by involving family experts to examine the relevance, the representativeness, and the clarity of the items. In the second phase, we validated the scale based on samples of fathers, mothers, and children aged 10 to 22 years of age in Chinese families in Hong Kong.

## 2. Materials and Method

### 2.1. Phase I Study: Development and Content Validation of the C-FRS

Based on Walsh’s family resilience framework [9], the first two authors developed an item pool containing 51 items to cover all nine dimensions (making positive meaning about adversity, positive outlook, transcendence and spirituality, flexibility, connectedness, social and economic resources, clear information about adversity, open emotional expression, and collaborative problem solving), with each dimension consisting of five to seven items. We paid particular attention to the indigenous Chinese context when developing the items. We then conducted a content validation of the C-FRS by inviting experts in social services to assess the relevance, representativeness, and clarity of items in the item pool.

### 2.2. Participants and Procedure

Sixteen social workers who provided social work service for children, adolescents, and their families were invited to participate in the content validation study. The participants were recruited from four non-governmental organizations (NGOs) in Hong Kong. Five of them were social work supervisors, three participants were school social workers, three participants worked in family social work service centers, and five participants worked in children and youth service centers. Among the participants, 15 (93.8%) were female. They were experienced social workers who had worked in children, youth, and family services for over 5 years. Nine participants (56.3%) had social work experience of over 20 years, five (31.3%) had social work experience between 11 and 20 years, and two (12.5%) had experience between 5 and 10 years. They were requested to examine the following: (1) relevance (i.e., whether the test items are relevant to the construct or the dimension related to the construct); (2) clarity (i.e., whether the wording and phases of the items are clear and concise), and (3) representativeness of the items to a particular content domain (i.e., how well the items represent the construct) using a structured questionnaire. The participants completed the questionnaire separately in an anonymous manner.

### 2.3. Measurement

A self-administered questionnaire was used to collect the views of the experts. The participants were informed of the definitions, the related literature, and the domains of family resilience, as well as the assessment instrument. They were requested to fill out the questionnaire based on their judgment. Regarding the “relevance” of the test items for the construct, a 4-point rating scale (1 = irrelevant; 2 = item needs revision or otherwise would no longer be relevant; 3 = relevant but needs minor amendment; and 4 = relevant) was used. If the participants rated “1” or “2”, they were required to write down the justifications. When they rated “2” or “3”, they were asked to suggest improvement for the items. Regarding the “clarity” of the items, a 4-point Likert scale (1 = very unclear, 2 = unclear, 3 = clear, and 4 = very clear) was used. Again, recommendations for modification of wording and phases were requested when an item was perceived as unclearly presented. To evaluate how well the items would represent the domains, a 4-point Likert scale (1 = very inadequate, 2 = inadequate, 3 = adequate, and 4 = very adequate) was used. When the participants suggested that the items are inadequate to represent the domain, they were asked for an explanation about their view.

### 2.4. Data Analysis

We analyzed the content validity with the following two strategies: (a) computation of content validity index (CVI) on the relevance, clarity, and representativeness, and (b) qualitative recommendations of experts on improving the items. While the CVI of each item was calculated by dividing the number of experts who rated positively (three or four) on the item by the total number of experts, the CVI for the measure was calculated by averaging the CVI across the items within the measure. A CVI of 0.80 was recommended as supporting the content validity for a new measure [29].

## 3. Results and Discussion

Regarding relevance of the item to the construct, the CVI_(relevance)_ of each item ranged from 0.69 to 1.00. The overall CVI_(relevance)_ was 0.94, with 41 out of the 51 items showing a CVI_(relevance)_ of over 0.80. Five of the items (Items 3, 8, 19, 21, and 24) showed values of less than 0.80 (Table 1). Regarding the clarity of the items, the CVI_(clarity)_ of each item ranged from 0.63 to 1.00, with an overall CVI_(clarity)_ of 0.90. While 41 out of the 51 items showed a CVI_(clarity)_ of over 0.80, 10 of the items (Items 3, 17, 19, 21, 24, 36, 37, 38, 39, and 40) were less than 0.80. For all of the domains, the CVI_(representativeness)_ were over 0.80, ranging from 0.81 to 1.00, with an overall CVI_(representativeness)_ of 0.93. A summary of the qualitative comments and decisions are listed in Table 1. As most of the items could be modified, we decided to retain all 51 of the items for validation.

The content validation findings provide support for most of the items in the initial item pool based on the cutoff criteria of 0.80, which was proposed by Davis [29]. This study is very important because no study has been published on the content validation of family resilience measures. Hence, our effort is pioneering in the field of family resilience.

## 4. Phase II Study: Validation of the C-FRS

At this phase, we validated and refined the 51-item C-FRS. The father, the mother, and a child aged 10 to 22 years within a family were invited to be the respondents in the validation study. Three psychometric properties of the assessment tool were assessed, including the reliability, the convergent validity, and the factorial validity among the different family members (fathers, mothers, and children). Regarding the reliability, internal consistency was used based on Cronbach’s alpha and inter-item correlation coefficients [30]. Regarding the validity, convergent validity (with family functioning as the criterion) and factorial validity (EFA and CFA) were examined. While EFA provides exploratory evidence of a conceptual model [31], CFA serves to confirm the proposed conceptual model [32]. This two-step analytic approach has been widely used to establish the factorial validity of an instrument [33,34,35]. Regarding the convergent validity, given that family resilience was positively associated family functioning [36] and communication [37] in previous studies, it was hypothesized that the mean C-FRS scores would be positively associated with the mean scores of family functioning that were reported by the father, the mother, and the adolescent participants (Hypotheses 1a, 1b, and 1c), respectively.

For the measurement invariance, configural invariance (i.e., invariance of the factor pattern), metric invariance (i.e., invariance of the factor loadings), scalar invariance (i.e., invariance of the intercepts), invariance of factor variance, and factor covariance of the measurement across the father’s sample, the mother’s sample, and the children’s sample were performed.

### 4.1. Participants and Procedure

We recruited 1020 Chinese families (*N* = 2858) in Hong Kong with the help of NGOs and tertiary institutions. The NGOs provided the family social work service, the children and youth services, and the school social work service in Hong Kong. They helped us to identify the service users from their membership lists and invited family members to participate in this study. The two inclusion criteria were as follows: (a) families with Chinese ethnicity, and (b) those with a child or an adolescent aged between 10 and 22 years. If the participating family had more than one eligible child, the elder child was invited to participate because they could comprehend the questionnaire better. A supermarket coupon (with a value of HKD 250, which was roughly equal to USD 32) was given to each participating family as a compensation of their time and transportation expenses.

The mean ages of the fathers, the mothers, and the adolescents in the sample were 51.2 (*SD* = 8.3), 46.6 (*SD* = 7.0), and 16.4 (*SD* = 4.7), respectively. There were 462 (45.3%) male adolescents and 556 (54.5%) female adolescents (2 of the participants did not disclose their gender). Regarding the participants’ family status, 818 (80.2%) were intact families, 29 (2.8%) were single-father families, and 173 (17.0%) were single-mother families. The majority of the families (*n* = 504; 49.4%) earned a monthly household income of between HKD 10,001 (USD 1282.2) and HKD 30,000 (USD 3846.2), and 375 (36.8%) of the families had monthly household income of over HKD 30,000 (USD 3846.2). There were 125 (12.3%) families receiving financial assistance (i.e., Comprehensive Social Security Assistance) from the Government.

Trained social workers or researchers introduced the research to all of the family members. Written informed consent of all of the family members was obtained. The participants were invited to respond to a questionnaire including measures of family resilience, family functioning, and demographic characteristics. The data collection was performed either in social service units or in the participants’ homes, depending on the participants’ preference. The family members filled out the questionnaire in a self-administered format separately, in order to safeguard confidentiality. The participants put the completed questionnaire into a sealed envelope and returned it to the social workers/researchers. This study followed the ethical standards of the Human Subjects Ethics Sub-committee of an internationally recognized University and the 1964 Helsinki declaration and its later amendments or comparable ethical standards.

### 4.2. Measures

*The Chinese family resilience scale (C-FRS).* Based on the initial findings, we used the newly developed 51-item C-FRS with nine subdomains (i.e., meaning making, a positive outlook, transcendence and spirituality, flexibility, connectedness, social and economic resources, clear information about adversity, open emotional expression, and collaborative problem solving). The respondents rated each item on a six-point Likert scale ranging from one = “totally disagree” to six = “totally agree”. Higher mean scores indicate higher levels of family resilience. The C-FRS showed excellent internal consistency in this study (Cronbach’s alphas of fathers: 0.98, mothers: 0.98, and children: 0.98).

*The Chinese family assessment instrument (C-FAI).* The C-FAI that was adopted in the present study was a nine-item instrument [38] that was selected from the original 33-item version, which was developed by Shek [39]. It contains three stable reliable dimensions in the construct of family functioning, namely communication, mutuality, and harmony. A sample item reads “Family members support each other”. The respondents were asked whether their family resembled the situation that was described by the item and rated their responses on a five-point Likert scale ranging from “very dissimilar” to “very similar.” A higher mean score indicates a higher level of family functioning. The C-FAI that was reported by the fathers, the mothers, and their children showed good internal consistencies (Cronbach’s alphas of fathers: 0.89, mothers: 0.90, and children: 0.90).

### 4.3. Data Analyses

We first split the data randomly into two halves (Subsets A and B). While Subset A was used for testing EFA, Subset B was used for testing CFA [34]. We first conducted EFA in order to explore the factor structures of the C-FRS using IBM SPSS 26 software (IBM, Armonk, NY, USA). Principal axis factoring (PAF) extraction with a direct oblimin rotation (δ = 0) was used to determine the factor structure [40,41] of the C-FRS. The following two well-known criteria for determining the number of components were considered: Kaiser’s [42] criterion to retain eigenvalues that are greater than 1 (K1) and Cattell’s [43] scree test. In order to confirm the proposed factor structure, CFA based on structural equation modeling (SEM) was further performed using AMOS 26. We adopted the following goodness-of-fit indictors to evaluate the adequacy of models: the comparative fit index (CFI) and the Tucker–Lewis index (TLI) of greater than 0.90 for an adequate model; root mean square error of approximation (RMSEA) of smaller than 0.06 for a good fit model, and between 0.06 and 0.08 for an acceptable model fit [44]. After testing the factor structure of the C-FRS, we examined the higher-order factor structure of the tested model by CFA. As recommended by Hu and Bentler [44], when a higher order model fits the data, the higher order model is preferred as the final model, as it contains a parsimonious factor structure for the construct.

In order to examine the factorial invariance of the C-FRS across the family members (fathers, mothers, and children), we performed multiple group factor analyses in this study by adopting a mean and covariance structures analysis (MACS) approach [45,46]. First, we assessed the configural invariance (i.e., free from any constraints; Model 0). The first-order factor loading invariance was then evaluated (i.e., the equality constraints were imposed on first-order factor loadings; M1). We compared Model 0 and Model 1 using the indicators of model invariance that were suggested by Cheung and Rensvold [47], i.e., the non-significant chi-square difference and change in CFI is less than 0.01. Next, we assessed the second-order factor loading invariance (i.e., the equality constraints on the first- and second-order factor loadings were imposed; Model 2). Again, we compared Model 2 and Model 1 using indicators that were suggested by Cheung and Rensvold [47]. Then, we tested the different nested models subsequently, including the invariance of the intercepts of the measured variables (i.e., constraining the first- and second-order factor loadings and the intercepts of the measured variables to be equal across the groups; Model 3), the invariance of the intercepts of the first-order latent factors (i.e., constraining the first- and second-order factor loadings, and the intercepts of the measured variables and first-order factors to be equal across the groups; Model 4), the invariance of disturbances of the first-order factors (i.e., constraining the first- and second-order factor loadings, intercepts, and disturbances of the first-order factors to be equal across the groups; Model 5), and the invariance of the residual variance of the observed variables (i.e., constraining the first- and second-order factor loadings, intercepts, disturbances of the first-order factors, and the residual variances of the measured variables to be equal across the groups; Model 6) [46]. Again, the indicators that were suggested by Cheung and Rensvold [47] were used for the model comparison.

## 5. Results

### 5.1. Preliminary Analysis

The descriptive characteristics of the 51-item C-FRS from the full dataset are listed in Table 2. The mean values of all of the items ranged from 3.05 to 4.52. The skewness and the kurtosis indices of the items ranged from −0.63 to 0.13 and from −1.12 to 0.66, respectively, suggesting normal distributions of the data [48].

### 5.2. Exploratory Factor Analysis

A principal axis factoring (PAF) with direct oblimin rotation was conducted using Subset A (*N* = 1436) in order to explore the underlying factor structure of the 51-item C-FRS. The initial solution yielded six components with eigenvalues exceeding one, accounting for a total of 66.98% of the variance. An inspection of the scree plot also supported the retention of six factors. In terms of our theoretical nine-factor structure, “connectedness”, “open emotional expression”, and “collaborative problem solving” were combined to form one factor, explaining 54.21% of the variance (Table 3). The second factor was a combination of “meaning of adversity”, “positive outlook”, and Items 13 and 14, explaining 4.85% of the variance (Table 3). The third factor was “social and economic resources” (Items 33–37) and Item 15, explaining 2.65% of the variance (Table 3). The fourth factor was “clear information about adversity” (Items 38–41), explaining 1.99% of the variance (Table 3). The fifth factor consisted of “flexibility” (Items 20–25) and two items of “transcendence and spirituality”, explaining 1.74% of the variance (Table 3). Finally, the sixth factor comprised three items of “connectedness” (Items 26–28), explaining 1.55% of the total variance (Table 3). From the findings of the EFA, only six out of nine factors based on the theoretical framework of family resilience [9] were extracted. Furthermore, the dimensions of “transcendence and spirituality” was split into two factors, with one factor combining with “flexibility” and the other merging with “positive meaning of adversity” and “positive outlook” (Table 3). Similarly, the dimension of “connectedness” was also split into two factors, with one factor combining with “open emotional expression” and “collaborative problem solving”, and the other standing out separately (Table 3). Moreover, seven of the items (Items 15, 16, 17, 19, 26, 32, and 37) had a factor loading of less than 0.40. If we deleted all of the items at this stage, the dimension of “transcendence and spirituality” (Items 15–17 and 19) would be removed. Hence, we decided to delete Items 26, 32, and 37, and retain Items 15, 16, 17, and 19, at this stage. Hence, a 48-item measure was tested for performing CFA.

### 5.3. Confirmatory Factor Analyses

The CFA was conducted using the data of Subset B (*N* = 1422). The expectation-maximization (EM) method was used to handle the missing data [49]. As the skewness and the kurtosis values of all of the items were less than two and seven, respectively (Table 2), the assumption of multivariate normality was supported [50]. Thus, we used the maximum likelihood approach in conducting the CFA. We tested the one-factor model and the nine-factor model based on Walsh’s Family Resilience Framework [9]. The one-factor model did not fit the data well, with CFI = 0.764, TLI = 0.753 (<0.90), and RMSEA = 0.103 (>0.08) (Table 4). Regarding the nine-factor model, the results of the CFA of the 48-item C-FRS fitted the data well, with values of CFI = 0.925, TLI = 0.919 (<0.90), and RMSEA = 0.059 (Table 4). In order to improve the conciseness of the scale, several items were deleted with justifications. First, Item 15 had a factor loading of 0.28 (<0.40 [51]). Second, Items 8, 13, and 20 showed cross-loadings with other factors (high modification indices (MI) of covariance between the item and the other factor [52]), with MI in covariance of Item 8 and “meaning with adversity” = 100.00, that of Item 13 and “positive outlook” = 55.99, and that of Item 20 and “transcendence and spirituality” = 91.87. Third, Items 1 and 2 (MI = 230.80, residual correlations (RC) = 0.21), 30 and 31 (MI = 301.46, RC = 0.33), 50 and 51 (MI = 180.80, RC = 0.13) had a high MI in covariance that was greater than 100 and residual correlations that were greater than the absolute value of 0.10, which indicated mis-specifications [53]. We retained Items 16 and 50 (higher factor loadings in the corresponding domain) in order to make sure that we had an adequate number of items in each domain. Lastly, as Items 36 and 42 showed relatively lower factor loadings, we decided to delete the two items in order to enhance the conciseness and the parsimony of the measurement. After deleting thirteen items, we tested the 35-item C-FRS by conducting CFA.

The 35-item C-FRS showed a good fit of the data, with values of CFI = 0.969, TLI = 0.964 (>0.90 [44]), and RMSEA = 0.046 (<0.06 [44]) (Table 4). The factor loadings of the items ranged from 0.63 to 0.93 (Table 5). Thus, the findings supported a nine first-order factor structure.

### 5.4. Hierarchical Factor Analysis

Based on the theoretical framework of family resilience that was proposed by Walsh [9], we further tested the second-order factor model (i.e., the nine first-order factors subsuming in three second-order factors of “family beliefs systems”, “organizational patterns”, and “family communication”; [9]) by performing hierarchical factor analysis. The hierarchical factor structure of the C-FRS showed a good fit of data, with CFI and TLI values of 0.963 and 0.960, respectively (>0.90 [44]), and an RMSEA value of 0.049 (<0.06 [44]; Table 4). The factor loadings of “meaning making”, “positive outlook”, and “transcendence and spirituality” on “family beliefs system” were 0.88, 0.95, and 0.98, respectively (Table 5). The three primary factors of “flexibility”, “connectedness”, and “kin, social, and economic resources” also corresponded well to “organizational patterns”, with factor loadings of 0.89, 0.88, and 0.65, respectively (Table 5). The last three primary factors of “clear information about adversity”, “open emotional expression”, and “collaborative problem solving” represented “family communication” well, with factor loadings of 0.88, 0.92, and 0.98 respectively (Table 5). The results showed a hierarchical factor structure supporting Walsh’s Family Resilience Framework [9]. The nine subdimensions, three dimensions, and the overall scale were correlated with each other, with Pearson’s *r* ranging from 0.42 to 0.94 (Table 6). The 35-item C-FRS is listed in Table A1.

### 5.5. Measurement Invariance of the C-FRS across Family Members

Multiple group analyses were performed in order to examine whether there was invariance of the hierarchical factor models of the C-FRS across the father, the mother, and the child participants [46]. The unconstrained model showed a good data fit, with a value of CFI = 0.955 (>0.90 [44]) and RMSEA = 0.031 (<0.06 [44]; Table 7), indicating that the factor pattern was invariant across the family members. When configural invariance (Model 0) was assumed, we then tested the first-order factor loading invariance (Model 1). It was common to find that the Chi-square difference value was significant among the three groups (Δχ2 = 71.061, *p* < 0.001), as the likelihood ratio test is sensitive to large sample size [33]. The change in the CFI value among the father, the mother, and the child groups was 0.000, supporting first-order factor loading invariance across the family members (ΔCFI < 0.01 [47]). When first-order factor loading invariance was supported, second-order factor loading invariance across the family members (Model 2) was then tested. The ΔCFI between Models 2 and 1 was 0.002 [47], supporting second-order factor loading invariance of the C-FRS. The results also showed invariance of intercepts of the measured variables (Model 3), with ΔCFI between Models 2 and 3 of 0.000. The invariance of the intercepts of the second-order factors (Model 4) was also supported, with ΔCFI between Models 3 and 4 of 0.001. There was invariance on disturbances of the first-order factors (ΔCFI between Models 4 and 5 was 0.008). However, there was a difference across the family members in residual variances of the measured variables (Model 6), with ΔCFI = 0.944 between Models 5 and 6 (ΔCFI > 0.01 [47]). However, as the invariance of factor loadings and intercepts are more relevant when evaluating factorial invariance of a measure between different groups [54], the hierarchical factor model of the C-FRS was considered to be invariant among the father, the mother, and the child participants (Table 7).

### 5.6. Convergent Validity

As predicted, the 35-item C-FRS was positively associated with family functioning (C-FAI [39]) for the father (*r* = 0.67, *p* < 0.001), the mother (*r* = 0.68, *p* < 0.001), and the child (*r* = 0.73, *p* < 0.001) participants, thus supporting Hypothesis 1a–c.

### 5.7. Internal Consistency

The 35-item C-FRS showed good internal consistency, with a Cronbach’s alpha value of 0.98 and inter-item correlations of 0.56 (Table 6). The Cronbach’s alpha values of the second-order factors of the family beliefs system, the organizational patterns, and communication were 0.95, 0.92, and 0.96, respectively, and the inter-item correlations were 0.62, 0.56, and 0.68, respectively. The first-order subscales also showed good internal consistency, with Cronbach’s alpha values ranging from 0.79 to 0.94, and those of inter-item correlations ranged from 0.61 to 0.83. Table 6 shows Cronbach’s alpha and inter-item correlation values of the measures and their subscales.

Overall, the findings have provided support for the convergent validity, the factorial validity, and the internal consistency of the measure. Most importantly, the findings provide support for Walsh’s model and the measure was stable in the father, the mother, and the child samples in this study.

## 6. Discussion

This study examined the psychometric properties (the convergent validity, the factorial validity, and the internal consistency) of the Chinese family resilience scale (C-FRS). There are four unique attributes of this study. Primarily, this is a study in a non-Western context, with a particular focus on Chinese people. Although family researchers have paid increasing attention to family resilience in the Chinese context (e.g., [18,20]), researchers have commonly used translated family resilience measures that were developed in Western societies [13]. In our study, although we used Walsh’s [9] conceptualization of family resilience as the theoretical framework, the items were indigenously developed, which fitted the Chinese culture, where familism and collectivism are emphasized [55]. For instance, rather than focusing on self-differentiation and individualism, which are based on an individualistic orientation (e.g., a sample item of Sixley’s FRAS reads “We can deal with family differences in accepting a loss”), the C-FRS focuses on family solidity and interdependence among family members (e.g., a sample item of the C-FRS reads “Family members are united”). Furthermore, we invited experts in the social work field to rate the items in terms of their relevance, their representativeness, and their clarity (i.e., content validity), and give comments on how the items could be improved. The findings showed that the C-FRS is culturally fit for assessing the family resilience in Chinese communities.

Second, this study provides support for Walsh’s model consisting of three domains and nine dimensions. Our findings have shown that the 35-item C-FRS showed a hierarchical factor structure with nine first-order factors subsumed under three second-order factors, namely the following: family beliefs system (meaning making, a positive outlook, and transcendence and spirituality), family organizational patterns (flexibility, connectedness, and social and economic resources), and family communication (clarity, open emotional expression, and collaborative problem solving); thus supporting the theoretical structure that is proposed by Walsh’s family resilience framework [9]. In contrast to the observation that results that are based on family resilience scales are not consistent with Walsh’s [9] conceptualization of family resilience in local and global contexts [24], the C-FRS fits Walsh’s family resilience framework well. The scale and its subscales are valid and reliable in assessing family resilience and its corresponding components in the present study. The previous Chinese studies did not provide support for Walsh’s model, which was likely because of two reasons. First, they did not employ large samples. Second, the translated measures may not be able to capture the characteristics of Chinese families.

Moreover, the hierarchical factor model offers a parsimonious structure on how first-order factors are interrelated into meaningful patterns [33]. Besides, hierarchical factor analysis removes random measurement error of the first-order factors and suggests that the variance of the second-order factors can be explained by the first-order factors [56]. Measurement invariance tests to assess the invariance of the C-FRS across different members in a family is also a methodological advance in this study, which encourages more family-based research to be conducted using multiple data sources.

Third, we used multiple informants to provide support for the invariance of the factor structure across fathers, mothers, and adolescent children. Instead of just focusing on an individual perspective, we endorsed a family systems perspective [25,26] and recruited the father, the mother, and a child/adolescent within a family to fill out the questionnaire. This is critically important for a family assessment tool because each family member contributes to the family processes and wellbeing. A measurement that can adequately assess the characteristics and the patterns of family processes and strengths among family members is important in order to capture the different perspectives from the different family members. Unfortunately, previous research studies employed only a single source of informant [17,18], which restricts the development of family resilience research adopting a family-based perspective and involving different family members.

In this study, the measurement invariance tests showed that the scale was invariant among the fathers, the mothers, and the children/adolescents, suggesting that each family member shares similar interpretations about the characteristics and the patterns of family resilience. Moreover, as the participants were mainly recruited from the community and the sample size was considered to be large in a validation study, the 35-item C-FRS can be widely used to assess the family resilience of Chinese families in facing global external threats (e.g., the COVID-19 pandemic, economic downturn, etc.). The scale and its subscales can help us to identify the strengths and the wellbeing of the families in facing crises and adversities, which is important for formulating social policies and designing appropriate social services that can buffer the negative impacts during the post-crisis stage.

Despite its pioneering nature, there are several limitations of this study. First, the findings of the EFA did not provide support for Walsh’s model. This is reasonable because EFA commonly assumes that measurement errors are uncorrelated with each other [57]. Secondly, although the literature points out that residual invariance is less relevant in testing the factorial invariance of a measurement tool between groups (e.g., [54]), further studies on the difference in residuals of measured factors of the C-FRS is suggested. Third, as we collected the data during the COVID-19 pandemic, the responses may have been influenced by different forms of adversities and hardship that different family members might have encountered, such as infections, loss of beloved ones, social isolation, and financial insecurity [58,59]. Nevertheless, the factorial invariance findings are robust across the different informants within the family. Fourth, we collected data from families with children and adolescents who were aged between 10 and 22 years, which covered a large age range of children and adolescents. As younger children may have difficulties in understanding the complex concepts of family resilience, we should explore this issue in future studies. Finally, our study was conducted using a Hong Kong Chinese family sample. It is recommended to replicate the current research in other Chinese communities (e.g., American Chinese, Mainland China, etc.,) and some Asian countries sharing similar cultural features (e.g., Japan and Singapore) [33].

## 7. Conclusions

Despite the limitations, the present study underscores the need to develop an indigenous family resilience scale based on the Chinese cultural characteristics, emphasizing familism, collectivism, and interdependence [23]. Besides, the content validation was performed by recruiting family social workers in order to assess the relevance, the representativeness, and the clarity of the items corresponding to the components of family resilience. Furthermore, this study used a large sample of multiple family members to assess the reliability, the concurrent validity, and the dimensionality of the measure. The findings suggest that the 35-item C-FRS is a valid and reliable measurement that objectively measures family resilience in Chinese communities. The C-FRS supports Walsh’s [9] theoretical conceptualization of family resilience by indicating a hierarchical factor structure of nine first-order factors, which are subsumed under three second-order factors, namely the following: family beliefs system (meaning making, a positive outlook, and transcendence and spirituality), family organizational patterns (flexibility, connectedness, and social and economic resources), and family communication (clarity, open emotional expression, and collaborative problem solving). Moreover, the C-FRS showed good internal consistency, convergent validity, and factorial invariance across the family members. Thus, the scale showed good psychometric properties in assessing family resilience among fathers, mothers, and child/adolescents in Chinese families. In view of a strong demand for, but a dearth of validated instruments that objectively measure the family resilience among different family members in Chinese contexts, our study takes a pioneering step by developing and validating the C-FRS that can be objectively used to assess family resilience in Chinese communities.

## Figures and Tables

**Table 1:** Modification of Items on the Chinese Family Resilience Scale (only those with modification are highlighted).

Item No.	Items	CVI		Problems and Suggestions Made	Modifications Suggested (Revised Item)
		Relevance	Clarity		
3	Family members believe that “the bitter one can taste, the higher rank one can be.” (A Chinese proverb).	0.69	0.69	Difficult to understand as it is a proverb The wording is too hard to understand	Family members believe that one can develop through pain.
8	Family members believe that “there should be hope behind the dark cloud.”	0.75	0.81	Difficult to understand as it is a metaphor Prefer to use a common saying: “there should be sunshine after the rain.”	Family members believe that no one can be free from adversity, thus one should learn how to face it with serenity.
17	Family will become creative when facing difficulties, as they know how to set set priority.	0.88	0.75	The statement is unclear. What is the relationship between creativity and setting priority?	Family members will foster creativity in facing adversity.
19	Family members have similar beliefs and values.	0.63	0.69	Too abstract Too vague	Family members embrace shared faith and similar values.
21	We have procedures in managing crisis in my family.	0.69	0.69	Too vague It is unclear whether “procedure” or “division of labor” should be focused on	Our family has developed procedures for managing a crisis.
24	Our family structure allows flexibility for us to handle sudden changes.	0.75	0.69	There is no need to focus on family structure Unclear when family structure will allow flexibility	Family members work out flexibly to handle sudden changes during crisis.
36	Our community takes care of those families having difficulties.	0.94	0.75	Should the statement relate to the society instead of the community? It is better to use “will care”	Our community will give support to those families facing difficulties.
37	Our family members have financial resources to overcome a difficult time.	0.94	0.75	It is unclear whether saving, pension, or other financial resources are referred to	Our family members are financially sustainable to face a difficult time.
38	Family members will actively collect accurate information when facing crisis.	0.94	0.63	Unclear wording It is unclear whether accuracy of information is the focus	In times of crisis, our family will actively collect information and learn knowledge related to the crisis.
40	Family members will seek more information in order to solve the problems arisen from the crisis.	0.94	0.69	It is unclear whether information search or problem solving is the main focus	In times of crisis, family members seek clear information on solutions to the crisis.
41	In times of difficulties, family members will share their information.	1.00	0.69	It is better to use “information about adversity” rather than all kinds of information	In times of difficulties, family members share the collected information about the adversity.

**Table 2:** Descriptive Statistics of the Chinese Family Resilience Scale (Overall).

Dimension	Subdimension	Item No.	Chinese Family Resilience			
			*M*	*SD*	Skewness	Kurtosis
Family beliefs system	Meaning making	1	3.94	1.16	−0.40	0.14
		2	4.18	1.18	−0.49	0.14
		3	4.04	1.15	−0.45	0.27
		4	4.27	1.11	−0.55	0.45
		5	4.31	1.13	−0.60	0.48
		6	4.33	1.15	−0.56	0.36
		7	4.35	1.10	−0.56	0.47
	Positive outlook	8	4.30	1.15	−0.53	0.29
		9	4.12	1.16	−0.44	0.18
		10	4.15	1.18	−0.46	0.12
		11	4.09	1.13	−0.45	0.27
		12	4.18	1.06	−0.52	0.66
	Transcendence and spirituality	13	4.30	1.12	−0.57	0.52
		14	3.94	1.21	−0.34	−0.02
		15	3.08	1.59	0.13	−1.12
		16	3.99	1.20	−0.45	0.13
		17	3.89	1.17	−0.37	0.16
		18	4.18	1.12	−0.53	0.47
		19	3.96	1.21	−0.39	−0.01
Organizational patterns	Flexibility	20	4.05	1.08	−0.44	0.49
		21	3.72	1.15	−0.24	0.01
		22	3.94	1.09	−0.36	0.34
		23	3.96	1.08	−0.41	0.49
		24	3.96	1.09	−0.39	0.33
		25	4.10	1.10	−0.44	0.30
	Connectedness	26	4.26	1.13	−0.52	0.34
		27	4.43	1.15	−0.63	0.47
		28	4.52	1.12	−0.61	0.40
		29	4.44	1.18	−0.62	0.24
		30	3.91	1.35	−0.36	−0.47
		31	4.11	1.25	−0.45	−0.11
		32	4.31	1.16	−0.63	0.39
	Kin, social, and economic resources	33	3.85	1.28	−0.47	−0.13
		34	3.88	1.35	−0.46	−0.33
		35	3.05	1.43	0.10	−0.86
		36	3.47	1.33	−0.16	−0.51
		37	3.83	1.23	−0.35	−0.11
Communication processes	Clear information about adversity	38	3.97	1.13	−0.51	0.37
		39	3.94	1.10	−0.45	0.45
		40	4.06	1.09	−0.52	0.58
		41	4.06	1.10	−0.54	0.57
	Open emotional expression	42	3.90	1.22	−0.42	0.00
		43	3.89	1.19	−0.40	0.03
		44	3.89	1.22	−0.37	−0.07
		45	3.94	1.20	−0.34	−0.05
		46	3.77	1.23	−0.29	−0.14
	Collaborative problem solving	47	4.18	1.15	−0.52	0.42
		48	4.21	1.14	−0.58	0.54
		49	4.19	1.20	−0.51	0.11
		50	3.96	1.20	−0.39	0.01
		51	4.06	1.20	−0.51	0.19

**Table 3:** Initial Exploratory Factor Analysis (Subsample A).

Item	Chinese Family Resilience					
	Six-Factor Model with Eigenvalues > 1					
1	0.10	**−0.58**	0.06	0.04	−0.09	0.01
2	0.12	**−0.68**	0.02	0.00	−0.01	0.08
3	0.01	**−0.78**	0.02	−0.03	0.08	−0.03
4	−0.07	**−0.83**	−0.01	−0.12	0.05	0.10
5	−0.05	**−0.90**	−0.02	−0.08	0.07	0.07
6	−0.06	**−0.86**	0.04	−0.05	0.02	0.02
7	−0.03	**−0.83**	−0.04	−0.09	−0.04	0.02
8	−0.01	**−0.83**	0.07	0.05	0.03	0.01
9	0.12	**−0.66**	0.06	0.11	−0.10	−0.07
10	0.13	**−0.58**	0.11	0.14	−0.12	0.00
11	0.08	**−0.61**	0.05	0.12	−0.23	−0.08
12	0.08	**−0.60**	−0.01	0.02	−0.23	−0.02
13	0.08	**−0.65**	−0.01	0.07	−0.15	−0.01
14	0.09	**−0.51**	0.13	0.15	−0.21	−0.12
15	0.06	−0.11	**0.27**	0.13	−0.04	−0.07
16	0.26	−0.30	0.04	0.07	**−0.29**	−0.08
17	0.23	−0.29	0.10	0.11	**−0.35**	−0.16
18	0.06	−0.34	0.01	0.02	**−0.40**	−0.07
19	0.32	−0.18	0.03	0.09	**−0.37**	−0.02
20	0.13	−0.21	−0.03	−0.05	**−0.58**	−0.08
21	0.03	0.01	0.11	0.01	**−0.70**	−0.13
22	0.06	−0.01	0.07	−0.02	**−0.73**	0.09
23	0.02	0.01	0.03	−0.03	**−0.86**	0.06
24	0.01	−0.03	0.01	−0.08	**−0.80**	0.10
25	0.00	−0.06	0.09	−0.07	**−0.67**	0.20
26	0.21	−0.20	0.07	−0.04	−0.33	**0.35**
27	0.36	−0.19	0.02	0.01	−0.19	**0.45**
28	0.29	−0.20	0.04	−0.02	−0.18	**0.47**
29	**0.45**	−0.18	0.07	0.05	−0.09	0.40
30	**0.44**	−0.04	0.22	0.15	−0.09	0.18
31	**0.49**	−0.06	0.16	0.13	−0.16	0.26
32	**0.35**	−0.21	0.15	0.05	−0.12	0.31
33	−0.06	0.00	0.74	−0.06	−0.09	0.13
34	−0.04	−0.03	0.70	−0.10	−0.02	0.16
35	−0.01	0.04	0.76	0.04	−0.01	−0.10
36	0.19	−0.05	0.47	−0.03	0.10	−0.07
37	0.03	−0.04	0.34	−0.29	−0.22	0.01
38	0.20	−0.17	0.23	**−0.44**	−0.21	−0.05
39	0.29	−0.14	0.18	**−0.45**	−0.21	−0.07
40	0.29	−0.19	0.12	**−0.46**	−0.21	−0.05
41	0.39	−0.12	0.06	**−0.44**	−0.21	−0.04
42	**0.71**	0.01	0.05	−0.17	−0.01	−0.08
43	**0.84**	0.03	0.03	−0.06	−0.03	−0.07
44	**0.90**	0.01	0.01	0.03	0.02	−0.02
45	**0.92**	0.04	0.03	0.02	0.02	−0.04
46	**0.74**	0.00	0.09	0.10	−0.06	−0.04
47	**0.76**	−0.11	−0.01	−0.05	0.01	0.15
48	**0.68**	−0.13	0.02	−0.06	−0.04	0.13
49	**0.68**	−0.10	−0.04	−0.06	−0.06	0.16
50	**0.71**	−0.03	0.06	−0.01	−0.10	0.02
51	**0.69**	−0.05	0.03	−0.05	−0.10	0.09
% of Variance	54.21	4.85	2.65	1.99	1.74	1.55
Total variance (%)	66.98					

Note. Bold values are the highest loadings obtained by a variable among the factors.

**Table 4:** Goodness-of-Fit Indices for Confirmatory Factor Models and Hierarchical Factor Models of C-FRS (Subsample B).

Description		*χ^2^*	*df*	*χ^2^/df*	CFI	TLI	RMSEA
After EFA item deletion (48 items)							
	One-factor model	17314.999 ***	1080	16.032	0.764	0.753	0.103
	Nine-factor model—based on conceptual framework	6172.794 ***	1044	5.913	0.925	0.919	0.059
After CFA item deletion (35 items)							
	One-factor model	11887.055 ***	560	21.227	0.772	0.757	0.119
	Nine-factor model—based on conceptual framework Hierarchical factor structure—three second-order	2080.451 ***	524	3.970	0.969	0.964	0.046
		2393.186 ***	548	4.367	0.963	0.960	0.049

*** *p* < 0.001.

**Table 5:** Standardized Factor Loadings of Nine-Factor Structure Model and Hierarchical Factor Models of 35-item Chinese Family Resilience Scale.

Higher-Order Construct	Construct	Item	Nine-Factor Structure	Hierarchical Factor Structure Model	
			Factor Loading	First-Order Factor	Second-Order Factor
Beliefs system					
	Meaning making				0.91
		3	0.76	0.77	
		4	0.81	0.81	
		5	0.87	0.87	
		6	0.86	0.87	
		7	0.86	0.90	
	Positive outlook				0.96
		9	0.86	0.87	
		10	0.88	0.88	
		11	0.90	0.90	
		12	0.84	0.87	
	Transcendence and spirituality				0.98
		14	0.81	0.82	
		16	0.79	0.79	
		18	0.77	0.76	
		19	0.81	0.79	
Organizational patterns					
	Flexibility				0.90
		22	0.89	0.90	
		23	0.92	0.93	
		24	0.90	0.90	
		25	0.87	0.87	
	Connectedness				0.90
		27	0.93	0.94	
		28	0.93	0.93	
		29	0.87	0.88	
	Kin, social, and economic resources				0.65
		33	0.84	0.86	
		34	0.81	0.84	
		35	0.63	0.66	
Communication					
	Clear information about adversity				0.87
		38	0.83	0.86	
		39	0.87	0.87	
		40	0.91	0.92	
		41	0.87	0.89	
	Open emotional expression				0.91
		43	0.86	0.86	
		44	0.90	0.91	
		45	0.91	0.92	
		46	0.83	0.83	
	Collaborative problem solving				0.97
		47	00.90	0.91	
		48	0.90	0.91	
		49	0.85	0.88	
		50	0.86	0.86	

**Table 6:** Correlations of Chinese Family Resilience and its Subscales (Overall).

	*M*	*SD*	Cronbach’s Alpha	Inter-Item Correlations	Correlations												
					1	2	3	4	5	6	7	8	9	10	11	12	13
1. Family resilience	4.05	0.88	0.98	0.56		0.92	0.94	0.94	0.81	0.87	0.89	0.87	0.86	0.68	0.85	0.86	0.90
2. Family beliefs system	4.14	0.92	0.95	0.62			0.80	0.79	0.91	0.95	0.93	0.79	0.77	0.51	0.72	0.72	0.76
3. Organizational patterns	4.02	0.91	0.92	0.56				0.84	0.69	0.76	0.79	0.88	0.87	0.80	0.77	0.76	0.80
4. Communication	4.00	0.97	0.96	0.68					0.67	0.75	0.79	0.78	0.79	0.59	0.89	0.93	0.95
5. Meaning making	4.26	0.98	0.92	0.69						0.79	0.74	0.68	0.68	0.42	0.63	0.58	0.64
6. Positive outlook	4.14	1.02	0.92	0.74							0.84	0.74	0.72	0.48	0.67	0.68	0.72
7. Transcendence and spirituality	4.02	0.99	0.86	0.61								0.78	0.73	0.52	0.69	0.73	0.75
8. Flexibility	3.99	1.00	0.94	0.79									0.74	0.53	0.73	0.69	0.74
9. Connectedness	4.46	1.08	0.94	0.83										0.48	0.68	0.71	0.79
10. Kin, social, and economic resources	3.60	1.14	0.79	0.57											0.56	0.54	0.53
11. Clear information about adversity	4.01	1.01	0.93	0.78												0.71	0.77
12. Open, emotional expression	3.87	1.10	0.93	0.76													0.85
13. Collaborative problem solving	4.13	1.07	0.93	0.78													

Note. All correlation coefficients are significant with *p* < 0.001.

**Table 7:** Goodness-of-Fit Indices for Factorial Invariance of Second-Order Factor Model of Chinese Family Resilience Scale across Family Members (Overall).

Description	Model	*χ^2^*	*df*	CFI	RMSEA	Comparison	Δχ2	Δ	Δdf
Baseline model (i.e., configural invariance)	M0	6098.973 ***	1644	0.956	0.031				
First-order factor loadings invariant	M1	6169.912 ***	1696	0.955	0.030	M1 versus M0	70.939 ***	0.001	52
First- and second-order factor loadings invariant	M2	6429.765 ***	1766	0.953	0.030	M2 versus M1	259.853 ***	0.002	70
First- and second-order factor loadings, and intercepts of measured variables invariant	M3	6453.326 ***	1778	0.953	0.030	M3 versus M2	23.561 *	0.000	12
First- and second-order factor loadings, and intercepts of measured variables and first-order factors invariant	M4	6572.242 ***	1808	0.952	0.030	M4 versus M3	118.916 ***	0.001	30
First- and second-order factor loadings, intercepts, and disturbances of first-order factors invariant	M5	7453.118 ***	1878	0.944	0.032	M5 versus M4	880.876 ***	0.008	70
First- and second-order factor loadings, intercepts, disturbances of first-order factors, and residual variances of measured variables invariant	M6	101907.460 ***	1785	0.000	0.140	M6 versus M5	94,454.342 ***	0.944	93

* *p* < 0.05, *** *p* < 0.001.

## Data Availability

Datasets generated for this research are available upon request to the corresponding author.

## Appendix A

**Table A1:** 35-item Chinese Family Resilience Scale.

1.	Family members believe “adversities” will enhance one’s growth.
2.	Family members think that adversities are inevitable yet comprehendible.
3.	Family members believe that problems they face are temporary and will pass.
4.	Family members believe “there are always more solutions than problems”.
5.	Family members believe that crisis is incredible in one’s life, thus one should learn to face it with serenity.
6.	When facing adversities, family members can stay optimistic.
7.	Family members are hopeful about their future.
8.	Family members see family crises in a positive light.
9.	In adversity, our family accept the things we cannot change but act proactively to those we may.
10.	Family members believe “crisis leads our family to grow strong”.
11.	Family members share similar values and life purposes and help each other to overcome difficulties.
12.	Family members face crises with calm and peace.
13.	Family members embrace shared faith and similar values.
14.	When facing adversity, family members take different roles with flexibility.
15.	Our family deal with crises in a flexible way.
16.	Family members can respond to emergencies flexibly.
17.	If needed, family members can take up more tasks and responsibilities flexibly.
18.	Family members mutually support each other.
19.	Family members take care of each other.
20.	Family members unite as one.
21.	Our friends will offer help when we face difficulties.
22.	Our relatives will offer help when we face difficulties.
23.	Our neighbors will offer help when we face difficulties.
24.	In times of crisis, our family will actively collect information and learn knowledge related to the crisis.
25.	In times of adversity, family members have adversity-related information.
26.	In times of crisis, family members seek clear information on solutions to the crisis.
27.	In times of difficulties, family members share the collected information about the adversity.
28.	Family members can express feelings to and share thinking with each other clearly.
29.	Family members open up themselves and are willing to listen to each other.
30.	Family members share feelings with each other.
31.	Family members understand and would not blame each other.
32.	When encountering problems, our family will solve them together.
33.	Family members strive together to meet family challenges.
34.	All family members participate in major family decisions.
35.	Family members are adept at discussing how to solve problems.
